# Neoadjuvant therapy of sequential TACE, camrelizumab, and apatinib for single huge hepatocellular carcinoma (NEO-START): study protocol for a randomized controlled trial

**DOI:** 10.1186/s13063-024-08340-1

**Published:** 2024-07-19

**Authors:** Yun Hao, Fei Xie, Yongjie Zhou, Chuan Li, Xiaoyun Zhang, Junyi Shen, Minghong Yao, Xin Sun, Jin Zhou, Tianfu Wen, Wei Peng

**Affiliations:** 1 Division of Liver Surgery, Department of General Surgery, West China Hospital, Sichuan University, Chengdu, China; 2 Department of Hepatic Biliary Pancreatic Surgery, the First People’s Hospital of Neijiang, Neijiang, China; 3 Laboratory of Liver Transplantation, Key Laboratory of Transplant Engineering and Immunology, NHC, West China Hospital, Sichuan University, Chengdu, China; 4 Chinese Evidence-based Medicine Center, West China Hospital, Sichuan University, Chengdu, China

**Keywords:** Hepatocellular carcinoma, Neoadjuvant therapy, Camrelizumab, Apatinib, Transarterial chemoembolization

## Abstract

**Background:**

The high recurrence rate after liver resection emphasizes the urgent need for neoadjuvant therapy in hepatocellular carcinoma (HCC) to enhance the overall prognosis for patients. Immune checkpoint inhibitors, camrelizumab combined with an anti-angiogenic tyrosine kinase inhibitor (TKI) apatinib, have emerged as a first-line treatment option for patients with unresectable HCC, yet its neoadjuvant application in combination with transarterial chemoembolization (TACE) in HCC remains unexplored. Therefore, this study aims to investigate the efficacy and safety of sequential TACE, camrelizumab, and apatinib as a neoadjuvant therapy for single, huge HCC.

**Methods:**

This multi-center, open-label randomized phase 3 trial will be conducted at 7 tertiary hospitals. Patients with single huge (≥ 10 cm in diameter), resectable HCC will be randomly assigned in a 1:1 ratio to arm of surgery alone or arm of neoadjuvant therapy followed by surgery. In the neoadjuvant therapy group, patients will receive TACE within 1 week after randomization, followed by camrelizumab (200 mg q2w, 4 cycles), along with apatinib (250 mg qd, 2 months). Patients will receive liver resection after neoadjuvant therapy unless the disease is assessed as progressive. The primary outcome is recurrence-free survival (RFS) at 1 year. The planned sample size of 60 patients will be calculated to permit the accumulation of sufficient RFS events in 1 year to achieve 80% power for the RFS primary endpoint.

**Discussion:**

Synergistic effects provided by multimodality therapy of locoregional treatment, TKI, and anti-programmed cell death 1 inhibitor significantly improved overall survival for patients with unresectable HCC. Our trial will investigate the efficacy and safety of the triple combination of TACE, camrelizumab, and apatinib as a neoadjuvant strategy for huge, resectable HCC.

**Trial registration:**

www.chitr.org.cn ChiCTR2300078086. Registered on November 28, 2023. Start recruitment: 1st January 2024. Expected completion of recruitment: 15th June 2025.

## Introduction

### Background and rationale {6a}

Hepatocellular carcinoma (HCC) is one of the most prevalent and deadly cancers globally, especially in China, where HCC stands as the second most prominent contributor to cancer-related fatalities [1, 2]. It is generally accepted that complete extirpation of the tumor provides the best opportunity for a cure and offers a favorable overall survival (OS) for patients with resectable HCC [3–5]. However, HCC is considered a difficult disease to cure due to its high recurrence rate after surgery, which is believed to be associated with the liver microenvironment [6]. Effective and well-tolerated neoadjuvant therapies are urgently needed to prevent postoperative recurrence in order to improve the overall prognosis of patients with HCC.

Neoadjuvant therapy is commonly used for patients with malignancies to stage down advanced disease and reduce postoperative complications, however, there is insufficient research support and limited endorsement in major guidelines for neoadjuvant treatments for HCC [7]. It remains controversial whether transarterial chemoembolization (TACE), a potential neoadjuvant locoregional therapy for HCC, could reduce tumor recurrence and prolong survival or not. Results from some studies showed that neoadjuvant TACE did not improve surgical outcomes for resectable HCC [8]. Conversely, results from some other studies showed neoadjuvant TACE was associated with improved OS and recurrence-free survival (RFS) for huge HCC (≥ 10 cm) [9, 10]. On the other side, neoadjuvant TACE, inducing partial tumor necrosis, may result in less secure attachment of residual tumor cells, making them more prone to dislocation into the bloodstream during liver resection [8].

Tyrosine kinase inhibitors (TKIs) and immune checkpoint inhibitors (ICIs) are now commonly used as systemic therapy for HCC patients, which are also being incorporated into neoadjuvant treatments. Earlier research indicated that the combination of TKI and ICI produced a synergistic antitumor effect, concurrently promoting tumor immune stimulation and vascular remodeling [11, 12]. In the latest phase III clinical trial, the combination of camrelizumab and apatinib as a first-line treatment for unresectable HCC demonstrated a median OS time of up to 22.1 months with an objective response rate (ORR) of up to 34.3% [13]. This combination has also shown promising outcomes in neoadjuvant therapy. In a phase II trial, 94.4% of patients with Barcelona Clinic Liver Cancer (BCLC) B/C stage HCC successfully underwent surgery after neoadjuvant therapy of camrelizumab plus apatinib, with a 1-year RFS rate reaching 53.85% [14]. Besides, several studies showed the combination of locoregional and systematic treatments could significantly improve tumor response and OS for patients with advanced HCC [15–17]. Our prior study evaluated the safety and efficacy of a triple combination of TKI, TACE, and anti-PD-1 inhibitors for patients with unresectable HCC, and promising results were found [18]. Inspired by this, we tried to extend the use of the triple combination to adjuvant and neoadjuvant settings. A phase II trial of adjuvant therapy was initiated to investigate the efficacy of the triple combination in patients with a high risk of HCC recurrence after surgery [19]. Here, we designed a prospective, open-label, randomized phase 3 trial to investigate the efficacy of neoadjuvant therapy of sequential TACE, camrelizumab, and apatinib for a single huge HCC.

### Objectives {7}

The study aims to (1) directly compare the efficacy of neoadjuvant therapy followed by surgery and surgery alone on HCC recurrence risk and survival in patients with single huge HCC; (2) explore the safety of neoadjuvant therapy regimen of TACE, camrelizumab, and apatinib; and (3) offer evidence and guidance for neoadjuvant therapy strategy in HCC patients with a high risk of recurrence.

### Trial design {8}

The study is a prospective, multicenter, open-label, randomized controlled trial. Patients will be randomly assigned in a 1:1 ratio to either the experimental group (surgery after neoadjuvant treatment with TACE combined with camrelizumab and apatinib) or the control group (surgical resection without neoadjuvant treatment), using a parallel design with superiority.

## Methods: participants, interventions, and outcomes

### Study setting {9}

From January 2024 to June 2025, patients with single huge (≥ 10 cm in diameter) resectable HCC that are scheduled for liver resection will be screened for eligibility for this trial. Participant recruitment will be conducted at seven hospitals in Sichuan Province, including six general hospitals (West China Hospital, Sichuan Provincial People’s Hospital, Yibin Second People’s Hospital, Neijiang First People’s Hospital, Affiliated Hospital of Southwest Medical University, and Chengdu Third People’s Hospital) and one hospital specialized in oncology (Sichuan Cancer Hospital). All these hospitals are registered with the China National Medical Products Administration for conducting clinical trials. These hospitals are major healthcare institutions in southwestern China, thus patients enrolled represent to some extent the patient population of the region.

### Eligibility criteria {10}

#### Inclusion criteria

(1) Aged between 18 and 75 years old; (2) newly diagnosed with HCC following clinical diagnosis criteria of the American Association for the Study of Liver Diseases (AASLD) [20]; (3) single huge (≥ 10 cm in diameter) tumor without vascular invasion or extrahepatic metastasis; (4) adequate future liver remnnt (FLR) (for patients with liver cirrhosis, FLR more 40% of standard liver volume, for patients without cirrhosis, FLR more than 30% of standard liver volume); (5) adequate liver function: Child-Pugh A grade and indocyanine green retention test at 15 min (ICG-R15) < 20%; (6) adequate hematologic and vital organ function defined by laboratory test results obtained within 7 days before treatment; (7) for patients with hepatitis B virus (HBV) infection, HBV DNA < 2000 IU/ml during screening, initiation of anti-HBV therapy at least 14 days prior to neoadjuvant therapy and willingness to continue anti-HBV; (8) Eastern Cooperative Oncology Group (ECOG) performance status (PS) score ≤ 1.

#### Exclusion criteria

(1) Known cases of fibrolamellar HCC, sarcomatoid HCC, or mixed cholangiocarcinoma and HCC; (2) human immunodeficiency virus infection or autoimmune diseases; (3) a history of severe allergies to any monoclonal antibodies or investigational drug components; (4) currently participating in other clinical trials, or less than 30 days since the end of treatment with an investigational drug in a previous clinical study; (5) pregnant or lactating women; (6) other factors that could potentially affect study outcomes or lead to its early termination (alcohol, drug abuse, serious medical condition, etc.)

#### Criteria for study centers

(1) Academic hospitals with the availability of skilled in liver surgery, TACE, and systemic therapy for liver cancer; (2) desire and capacity to participate in the trial;

### Who will take informed consent? {26a}

Trained researchers will provide detailed study information, including design, aspects, benefits, and potential harms, to potential participants before enrollment.

### Additional consent provisions for collection and use of participant data and biological specimens {26b}

Enrolled participants will sign written informed consent, including the consent for collection and use of biological specimens, ensuring confidentiality of personal information.

## Interventions

### Explanation for the choice of comparators {6b}

Participants allocated to the arm of surgery alone will receive liver resection within 1 week after randomization according to existing guidelines [20]. We will collect the perioperative and follow-up data of participants allocated to the arm of surgery alone.

### Intervention description {11a}

Patients in the control arm will undergo liver resection within 1 week after randomization. Patients in the arm of neoadjuvant therapy will receive TACE within 1 week after randomization, followed by camrelizumab (200 mg q2w, for 4 cycles). They were also given oral apatinib mesylate tablets (250 mg qd, for 2 months). Patients must undergo imaging tests, including computed tomography (CT) scans of the chest/abdomen/pelvis, within 28 days before their first dose of medication. Additional scans of other body areas may be conducted if clinically necessary. After neoadjuvant therapy, a preoperative assessment including a CT scan will be conducted within 14 days (± 7 days) after the last dose of medication. Patients will receive surgery after neoadjuvant therapy if the disease is not assessed as progressive disease. To ensure the safety of the surgery, camrelizumab is discontinued for at least 2 weeks, while apatinib is discontinued for at least 1 week before the surgery.

All TACE procedures were performed by experienced physicians and a uniform treatment protocol was applied to each patient [21]. TACE was performed through the right femoral artery with local anesthesia. After arteriography of the celiac trunk and superior mesenteric artery to visualize arterial vascularization of the liver, body surface-dependent doses of the chemotherapeutic agents 5-fuorouracil (800~1000 mg) and epirubicin-adriamycin (30~40 mg) were injected. Subsequently, lipiodol and polyvinyl alcohol foam embolization particles were injected as selectively as possible into the hepatic segmental artery at the target tumor location. The embolization agent doses ranged from 5 to 30mL and were determined based on the tumor location, size, and number.

The dosages of apatinib and camrelizumab in this study will be selected based on recommended dosages in their drug labels and prior clinical studies for unresectable HCC [13].

### Criteria for discontinuing or modifying allocated interventions {11b}

Discontinuation or modifying of the allocated intervention is possible after a participant withdraws from the intervention and/or trial or based on clinical judgment by the intervention facilitator, e.g., when a participant is unable to continue due to adverse event (AE) caused by camrelizumab and/or apatinib.

### Strategies to improve adherence to interventions {11c}

Several strategies to improve adherence to interventions. Firstly, potential participants are informed of the potential benefits of neoadjuvant therapy based on present evidence. Secondly, potential participants are also informed of specific dates, cycles, and sites of intervention before they decide to participate in this trial. Thirdly, the trial covers the costs of medication, including camrelizumab and apatinib for neoadjuvant therapy. Fourthly, investigators and coordinators of the trial will schedule individual calls with each participant.

### Relevant concomitant care permitted or prohibited during the trial {11d}

All participants cannot receive any other neoadjuvant treatments outside of the trial. Steroid use is allowed when immune-related AE occurrs.

### Provisions for post-trial care {30}

Regular follow-up is planned in post-trial care. An insurance for this trial will cover the compensation to participants who suffer harm from trial participants if the AE is determined as treatment-related.

### Outcomes {12}

The primary endpoint is the 1-year RFS rate, defined as the time period from randomization to the first documented HCC recurrence or death from any cause. The key secondary endpoints include pathological remission degree and OS which was defined as the time period from randomization to death from any cause. Pathological complete remission (pCR) is defined as existent ≤ 1% active tumor cells, while major pathological remission (mPR) is defined as ≤10% active tumor cells. Safety outcomes encompass adverse events (AEs), defined as the occurrence of ≥ 3 grade hematological or non-hematological toxicities (including but not limited to liver dysfunction, hematological abnormalities, hypertension, diarrhea, proteinuria, hand-foot syndrome, etc.). The severity of adverse events will be graded according to the Common Terminology Criteria for Adverse Events (CTCAE v5.0).

### Participant timeline {13}

**Fig. 1:**
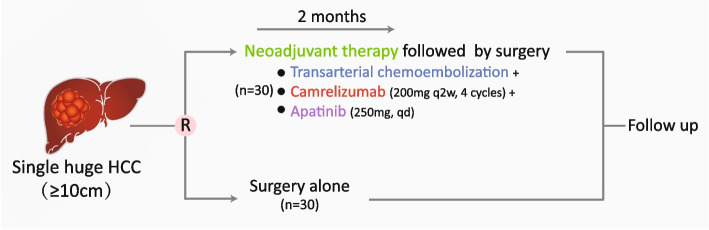
Participant timeline

Please see Fig. 1 for details of the participant timeline. Details of the schedule for enrolment, interventions, and assessments can be found in Table 1.

**Table 1:** Schedule of enrolment, interventions, and assessments

	**STUDY PERIOD**									
**Timepoint**	***−Day ***_***28***_	***Week 1***	***Week 3***	***Week 5***	***Week 7***	***Week 9***	***Month 3***	***Month 6***	***Month* 9**	***Month* 12**
**Visit window(days)**		***Day***_***1~7***_	***± 3***	***± 3***	***± 3***	***± 3***	***± 7***	***± 7***	***± 7***	***± 7***
**Enrolment:**										
***Eligibility screen***										
***Informed consent***	X									
***Baseline***	X									
***Laboratory test***	X									
***CT scans***	X									
***Allocation***	X									
**Interventions:**										
**control arm: *surgery alone***		X								
**Neoadjuvant arm: *TACE***		X								
***Camrelizumab***		X	X	X	X					
***Apatinib***		X	X	X	X					
**Safety assessments:**										
***Adverse events***		X	X	X	X	X				
***Living state***			X	X	**X**	**X**	X	X	**X**	**x**
**Assessments:**								X		
***Lab, CT, etc***						X				
***Primary endpoint***										**X**
***Secondary endpoint***							X			**X**

### Sample size {14}

The sample size was performed using the software PASS (version 2015). The calculation is based on the primary endpoint which is RFS in the present study. Previous research data showed that the 1-year RFS rate was 40% for patients with a single huge HCC after surgery [9]. Our prior study showed that the 1-year event-free survival (EFS) rate was 65% for patients with unresectable HCC after receiving a similar treatment regime [22]. Therefore, we hypothesize that neoadjuvant therapy could increase the 1-year RFS rate to 70% for patients with a single huge HCC. Using an alpha of 0.025 at one-sided, a power of 80%, a total of 60 participants needed to be enrolled with 30 in each arm when considering an attrition rate of 10% and an anticipated recruitment period of 1.5 years.

### Recruitment {15}

We aim to enroll a total of 60 participants across seven hospitals. To achieve this, monthly online conferences are scheduled to monitor enrollment progress at each hospital and strategize methods to accelerate recruitment. Participants at each center will be contacted via telephone. All seven centers are expected to collectively recruit approximately 3–4 participants per month, with an anticipated recruitment period of 1.5 years. The study will run from January 2024 to June 2026.

## Assignment of interventions: allocation

### Sequence generation {16a}

Participants will be randomly assigned to the arm of surgery alone or the arm of neoadjuvant therapy followed by surgery in a 1:1 ratio, using SAS statistical software.

### Concealment mechanism {16b}

The generated random allocation sequence will be sealed in sequentially numbered, opaque envelopes, with each participant’s random number remaining unchanged throughout the study. Envelopes will be opened only when eligible study subjects agree to participate.

### Implementation {16c}

The allocation sequence is generated by the trial statistician, MHY. Participants are enrolled by the study team and assigned to the intervention by the trial investigators.

## Assignment of interventions: blinding

### Who will be blinded {17a}

Data analysts are blinded to the interventions and will not participate in patient enrollment or medical care. It is not possible to blind trial participants, care providers, or investigators due to the complexity of the neoadjuvant therapy.

### Procedure for unblinding if needed {17b}

Since study team members are unblinded, members who participate in patient enrollment and trial intervention will not complete data collection or outcome assessments during the trial. As participants are not blinded, there is no further procedure for unblinding participants.

## Data collection and management

### Plans for assessment and collection of outcomes {18a}

All investigators and coordinators will receive specialized training for data collection. Baseline assessments will be conducted before randomization. Any AE during neoadjuvant therapy will be assessed and managed by investigators and recorded timely. Tumor response after neoadjuvant therapy will be performed via enhanced CT using the response evaluation criteria in solid tumors, version 1.1 (RECIST 1.1), and the modified response evaluation criteria in solid tumors (mRECIST) in 2 weeks after neoadjuvant therapy. Perioperative data, including intraoperative information and postoperative complications which will be graded by Clavein-Dindo criteria, will be recorded before discharge.

### Plans to promote participant retention and complete follow-up {18b}

Participants will receive ¥100 per trial visit as reimbursement for their time. If a participant chooses to withdraw from the trial, reasons for withdrawal will be recorded but no further outcome data will be collected.

### Data management {19}

Trial-related data will be recorded timely using the Electronic Data Capture (EDC) system by research staff only and password-protected access after necessary EDC training. A data manager will supervise routine accuracy checks and address any issues with investigators. Once the data are declared clean, the chief investigator must complete the signature panel associated with each subject and lock the data at the end of the trial. Hard-copy documents outside the EDC system will be stored securely, and electronic files will have password protection. All documents will be saved for at least 5 years post-publication, with original data available upon request to the chief investigator (CI).

### Confidentiality {27}

Personal information will remain confidential. Prior to consent, the name and contact details of the potential participant are shared with the research team and recorded separately. Each participant will be allocated a unique trial ID, and all data will be stored according to this trial ID.

### Plans for collection, laboratory evaluation, and storage of biological specimens for genetic or molecular analysis in this trial/future use {33}

Peripheral blood and specimens of the resected tumor and precancerous tissue will be freshly stored in liquid nitrogen for genetic analysis to screen potentially sensitive populations to neoadjuvant therapy.

## Statistical methods

### Statistical methods for primary and secondary outcomes {20a}

Primary efficacy will be assessed in all randomized patients using the intention-to-treat (ITT) principal. And the safety analysis will include all participants who received at least one post-randomization measure. Kaplan-Meier method is used to determine RFS and OS. Hazard ratios (HRs) with 95% confidence intervals (CIs) are estimated using Cox proportional risk models. Continuous variables are analyzed using either unpaired Student’s *t*-test or the Mann-Whitney *U* test, while categorical data are assessed using the chi-square test or Fisher’s exact test, as appropriate. Data analysis will be performed using SPSS version 26.0 and *P* values < 0.05 are considered statistically significant. The flow of participants screened by the trial and trial results will be reported according to Consolidated Standards of Reporting Trials (CONSORT).

### Interim analyses {21b}

Besides the final analysis, a mid-term analysis will be scheduled for this trial. The purpose of the mid-term analysis is about the safety and efficacy of the study. The scheduled time for mid-term analysis is set at 6 months after the last enrolled patient. If the RFS invalid hypothesis is rejected prior to teh final analysis, the trial might be discontinued.

### Methods for additional analyses (e.g., subgroup analyses) {20b}

No plan for additional analyses.

### Methods in analysis to handle protocol non-adherence and any statistical methods to handle missing data {20c}

As we target the treatment regimen estimate, we will include all participants in the primary analysis including those who do not adhere to the protocol. A supplementary analysis will be performed using a per-protocol principal to estimate the treatment effect for those who switch to another arm after randomization. Participants who have missing data points will be included in the analysis under a Missing at Random assumption implicit when using the longitudinal model.

### Plans to give access to the full protocol, participant-level data and statistical code {31c}

The full protocol is available from the Ethics Committee of West China Hospital, Sichuan University. The study team will retain the exclusive use of data until the publication of all planned analyses has been completed. An anonymized dataset and extracts of the statistical code will be available from the corresponding author upon reasonable request.

## Oversight and monitoring

### Composition of the coordinating center and trial steering committee {5d}

The research team involved in the day-to-day management of the trial meets weekly, while the wider study team including all sub-CIs at each partner hospital meets monthly, to discuss current issues and updates for the trial. The Trial Steering Committee (TSC) has been established based on internal criteria to oversee the progress and conduct of the trial. Membership of the TSC includes an independent chair, independent statistician, independent experts including surgeons, radiologists, oncologists, and representatives of the study team. The TSC met at the beginning of the trial and will meet quarterly during the trial.

### Composition of the data monitoring committee, its role and reporting structure {21a}

The Data Monitoring Committee (DMC) is fully independent which is selected by internal criteria and responsible for overseeing the safety of the trial. The DMC met at the start of the trial and will meet quarterly for the trial’s duration.

### Adverse event reporting and harms {22}

Any AE will be documented in case report forms (CRFs), with severe AE reported to the ethics and administrative department within 24 h.

### Frequency and plans for auditing trial conduct {23}

The trial will permit direct access to participants’ records and documents for the purposes of monitoring, auditing, or inspection by the Sponsor annually.

### Plans for communicating important protocol amendments to relevant parties (e.g., trial participants, ethical committees) {25}

All protocol amendments will be approved by the ethics committee before implementation. All protocol amendments will be communicated to partner hospitals who will also issue local approval.

### Dissemination plans {31a}

Study findings will be disseminated through national and international conferences, as well as peer-reviewed publications. A lay summary will also be produced for all participants. Trial register will be updated after the completion of recruitment.

## Discussion

Therapy for HCC remains a formidable challenge, particularly in cases involving extremely large tumors, multiple primary tumors, or major vascular invasion—referred to as high-burden tumors [23–25]. Our study focuses on patients with a single huge HCC, which is recognized as an independent risk factor contributing to an increased risk of initial extra-hepatic recurrence [9, 23]. There has been an ongoing debate regarding the clinical staging and treatment strategy for large HCC [26–28]. According to the National Comprehensive Cancer Network (NCCN) Guidelines, liver resection is the preferred treatment for patients with any size of resectable solitary tumor, provided there is adequate liver function and liver remnant without major vascular invasion. The China Liver Cancer (CNLC) staging criteria classify a single large HCC as stage Ib (early stage), and liver resection is also the preferred treatment [29]. In contrast, the BCLC staging system suggests that single large HCCs often present with tumor-related symptoms, and HCC with symptoms should be staged as advanced disease [4]. Guidelines from BCLC recommend systemic therapy rather than liver resection for such cases. In line with the views of many scholars, we also believe that large tumors should not be considered a contraindication for surgical removal, particularly when patients have undergone effective and safe neoadjuvant treatment [25, 30, 31]. This not only increases the rate of surgical resection but also reduces the likelihood of postoperative recurrence, allowing patients to derive survival benefits from tumor resection.

Currently, there is no widely accepted neoadjuvant therapy for HCC. Strategies of neoadjuvant use of locoregional treatment, systemic treatment, or in combination are being actively carried out. TACE, a widely employed locoregional treatment for intermediate-stage HCC, could effectively reduce tumor burden by targeted arterial embolization and directly killing tumor cells, but on the other side, the hypoxic microenvironment leading by TACE procedure triggers the upregulation of angiogenic factors such as vascular endothelial growth factor (VEGF) and fibroblast growth factor (FGF), thereby promotes tumor angiogenesis and metastasis [32]. This may partially explain why the effect of neoadjuvant TACE on the survival of resectable HCC is controversial [8, 9]. Combination with a multi-kinase inhibitor that targets VEGF receptors and FGF receptors, could partially address the above problem. LAUNCH trial revealed that a combination of lenvatinib and TACE could significantly improve clinical outcomes for patients with advanced HCC [17]. Furthermore, TACE was found to increase immunogenicity by inducing inflammation and releasing tumor-associated antigens, which could enhance the antitumor activity of immunotherapy [33]. Therefore, a combination of locoregional and systemic interventions is likely to be a viable approach for a neoadjuvant regimen for HCC. Emerald-1, a phase 3, randomized study investigated the synergistic effect of the triple combination of TACE, bevacizumab, and durvalumab. The results showed the triple combination therapy significantly improved PFS when compared to TACE for patients with unresectable HCC [34]. The finding was in line with our previous study in which better survival was observed in patients with advanced HCC who received triple combination therapy of lenvatinib, TACE, and anti-PD-1 inhibitor [18, 22]. In this study, we design a triple combination of camrelizumab plus apatinib combined with TACE as a neoadjuvant treatment. Camrelizumab, a high-affinity IgG4-kappa monoclonal antibody targeting PD-1, has been approved in China for the treatment of advanced HCC patients. Apatinib is a novel, small molecule, selective VEGF receptor-2 tyrosine kinase inhibitor. The CARES-310 study revealed a significant clinical benefit in PFS and OS with combination therapy of camrelizumab and apatinib versus sorafenib for patients with unresectable HCC [13].

Our study has several limitations. Firstly, the sample size is relatively small, even though the calculation was based on previous research. Secondly, all the participants included in this study are from Sichuan, China. The generalizability of the findings to other geographic locations and ethnicities remains to be validated.

In summary, this study aims to investigate the efficacy and safety of the triple combination as a neoadjuvant treatment for resectable huge HCC. Simultaneously, it seeks to address the controversies and deficiencies in the existing guidelines regarding the treatment approach for single huge (> 10cm) hepatocellular carcinoma.

## Trial status

The study is ongoing. Participant recruitment started on 1st January 2024 based on protocol version 3 (18th October 2023). The recruitment is expected to be completed before 15th June 2025.

## Data Availability

The anonymized datasets of the present trial will be available as de-identified data upon request from Wei Peng (pengwei@wchscu.edu.cn), beginning 12 months and ending 5 years after the primary publication and planned primary analysis following approval of a methodologically sound proposal and a signed data-sharing agreement.

## Abbreviations

HCC: Hepatocellular carcinoma
TKI: Tyrosine kinase inhibitor
TACE: Transarterial chemoembolization
RFS: Recurrence-free survival
OS: Overall survival
ICI: Immune checkpoint inhibitors
ORR: Objective response rate
AASLD: American Association for the Study of Liver Diseases
FLR: Future liver remnant
ICG-R15: Indocyanine green retention test at 15 min
ECOG: Eastern Cooperative Oncology Group
PS: Physical status
CT: Computed tomography
pCR: Pathological complete remission
mPR: Major pathological remission
AE: Adverse event
CTCAE: Common Terminology Criteria for Adverse Events
CRF: Case report form
EFS: Event-free survival
HR: Hazards ratio
CI: Confidence interval
BCLC: Barcelona Clinic Liver Cancer
NCCN: National Comprehensive Cancer Network
CNLC: China Liver Cancer
VEGF: Vascular endothelial growth factor
FGF: Fibroblast growth factor
CI: Chief investigator
CONSORT: Consolidated Standards of Reporting Trials
TSC: Trial Steering Committee
DMC: Data Monitoring Committee
